# Novel Tsg101 Binding Partners Regulate Viral L Domain Trafficking

**DOI:** 10.3390/v13061147

**Published:** 2021-06-15

**Authors:** Madeleine Strickland, David Nyenhuis, Susan M. Watanabe, Nico Tjandra, Carol A. Carter

**Affiliations:** 1 Biochemistry and Biophysics Center, National Heart, Lung, and Blood Institute, National Institutes of Health, Bethesda, MD 20892, USA; Strickland.Maddy@gmail.com (M.S.); David.Nyenhuis@nih.gov (D.N.); tjandran@nhlbi.nih.gov (N.T.); 2 Department of Microbiology & Immunology, Renaissance School of Medicine, Stony Brook University, Stony Brook, NY 11794, USA; Susan.Watanabe@stonybrook.edu

**Keywords:** Tsg101, UEV, HIV-1, ESCRT, RNA, ubiquitin, E2 enzyme, prazoles, anti-viral, L domain, herpesvirus

## Abstract

Two decades ago, Tsg101, a component of the Endosomal Sorting Complexes Required for Transport (ESCRT) complex 1, was identified as a cellular factor recruited by the human immunodeficiency virus type 1 (HIV-1) to facilitate budding of viral particles assembled at the cell periphery. A highly conserved Pro-(Thr/Ser)-Ala-Pro [P(T/S)AP] motif in the HIV-1 structural polyprotein, Gag, engages a P(T/S)AP-binding pocket in the Tsg101 N-terminal domain. Since the same domain in Tsg101 that houses the pocket was found to bind mono-ubiquitin (Ub) non-covalently, Ub binding was speculated to enhance P(T/S)AP interaction. Within the past five years, we found that the Ub-binding site also accommodates di-Ub, with Lys63-linked di-Ub exhibiting the highest affinity. We also identified small molecules capable of disrupting Ub binding and inhibiting budding. The structural similarity of these molecules, prazoles, to nucleosides prompted testing for nucleic acid binding and led to identification of tRNA as a Tsg101 binding partner. Here, we discuss these recently identified interactions and their contribution to the viral assembly process. These new partners may provide additional insight into the control and function of Tsg101 as well as identify opportunities for anti-viral drug design.

## 1. Introduction

The ESCRT-I factor tumor suppressor gene 101 (Tsg101) plays a well-established role in promoting efficient budding of enveloped viruses belonging to several different virus families. As such, it represents a potential target for development of anti-viral agents with broad efficacy. Tsg101 is designated as a ubiquitin (Ub) E2 variant (UEV) protein since it lacks the catalytic Cys residue required for conjugation and transfer of Ub. UEV proteins play key roles with E3 ligases in control of protein trafficking, stability and DNA replication, vital functions that viruses can harness for their own production. The discovery that Tsg101 interacts with the HIV-1 structural precursor polyprotein, Gag, linked the ESCRT machinery in human cells to viral egress and much is now known about the process ([1,2,3], reviewed in [4,5,6,7]). Interestingly, viruses may exploit the ESCRT machinery at different replication stages, e.g., the retrovirus HIV-1 utilizes ESCRT factors at late stages of particle assembly to bud from the plasma membrane; herpes viruses, including herpes simplex viruses (HSV) and Epstein–Barr virus (EBV), bud initially from the inner nuclear membrane and later undergoing a second envelopment in the cytoplasm before finally exiting through the secretory pathway. Although occurring at different replicative stages and in different locations, the HIV-1 and herpesvirus budding events are believed to be analogous. In both cases, the membrane-remodeling component of the ESCRT machinery (ESCRT-III) is critical for release of assembled particles into the extracellular space (HIV-1) or into the space between the inner and outer nuclear membrane (HSV, EBV). In the case of HIV-1, it is known that Tsg101 functions both as a scaffold for ESCRT-I formation and in recruitment of ESCRT-III [4,8]. In contrast, little to nothing is known regarding the events that occur prior to ESCRT-III action in the case of EBV; in the case of HSV, it is controversial [9].

The direct interaction of Tsg101 and P(T/S)AP L(ate) domain motifs in viral structural proteins is now well-established as critical for budding of HIV and several other enveloped viral pathogens (reviewed in [10,11]). L domains can assume other forms in viruses related to HIV and in other virus families, however, the very strictly conserved P(T/S)AP L domain motif encoded in the HIV-1 and -2 structural protein Gag is recognized only by Tsg101. Conversely, Tsg101, as far as is known, does not recognize other L domains. The interaction with P(T/S)AP is mediated through the cellular protein’s N-terminal ubiquitin (Ub) E2 variant (UEV) domain [1,2,3]. The UEV domain is given this designation because it resembles canonical Ub-conjugating (E2) enzymes structurally but is unable to catalyze Ub transfer as it lacks the active site Cys that forms a transient thioester bond with the C-terminus of Ub [12,13]. The active site region is thus considered vestigial. Tsg101 is a key component of the Endosomal Sorting Complex Required for Transport-I (ESCRT-I). As such, the protein functions in recognition of cargo entering the cell and in sorting it for delivery to various destinations in the cell interior or recycling back to the plasma membrane. The UEV domain mediates these Tsg101 functions through the P(T/S)AP- and Ub-binding functions. The region downstream of the UEV domain in Tsg101 interacts with other proteins, including the binding partners with which it forms ESCRT-I, a complex that functions with ESCRT-0, -II, -III, and the ATPase Vps4 in endocytic trafficking (reviewed in [4,6,14,15]). As the member on which the partners nucleate, Tsg101 thus controls ESCRT-I formation and plays an essential scaffolding and mechanical role in addition to functioning as a conduit to ESCRT-III [8]. This scaffolding role is critical to viral particle budding [14,15]. In addition to enabling viral proteins linked directly to it through P(T/S)AP to access ESCRT-III membrane scission activity, Tsg101 also functions in multivesicular body (MVB) biogenesis, exosome secretion, and cytokinetic abscission during mitotic exit. Its steady state level is very tightly controlled by the Tsg101-associated Ub ligase (TAL) that targets Lys residues in the C-terminal region of the Tsg101 protein for covalent modification [16]. Although much is now known about ESCRT structure and function in general, understanding of the individual ESCRT factors is relatively limited.

**Figure 1:**
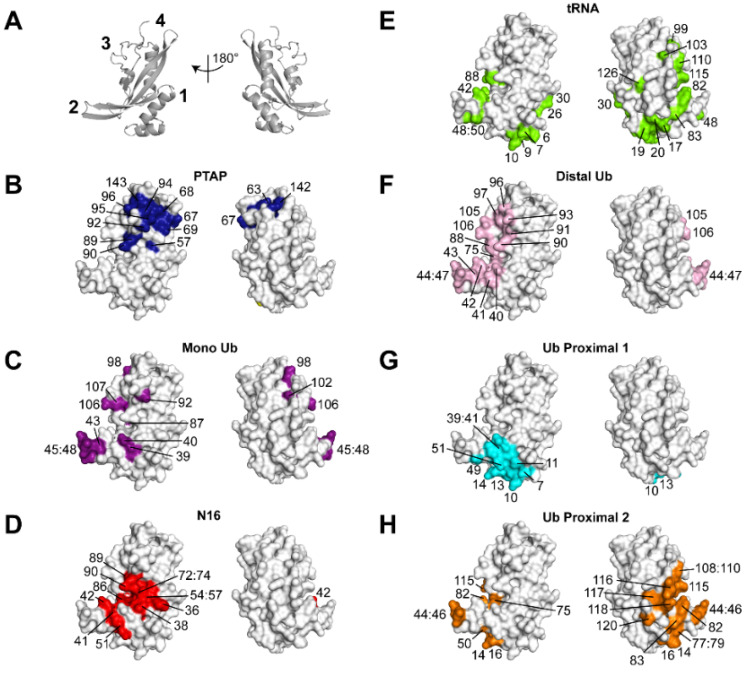
Binding capabilities of the Tsg101 UEV domain. Panel (**A**), UEV (gray) secondary structure elements (PDB ID: 1KPP [17]); numbers identify approximate locations of structural elements unique to the UEV domain compared to canonical E2 enzymes: 1, N-terminal α-helix-1; 2, β-hairpin; 3, vestigial active site; 4, P(T/S)AP-binding pocket. Panels (**B**–**H**), the same UEV structure (gray) is rendered in space-filling mode with recognition determinants of the indicated partner colored. Panels (**B**–**E**) highlight large chemical shift perturbations of Tsg101 measured by NMR of (**B**) a PTAP peptide, (**C**) monoubiquitin, and (**D**) N16, also known as tenatoprazole ((**B**–**D**), [23]), (**E**) tRNA [15]. Panels (**F**–**H**) highlight residues identified to be in (**F**) distal, (**G**) proximal site 1 and (**H**) proximal site 2 di-Ub binding sites and used as restraints for rigid-body docking and structure refinement.

Figure 1 summarizes the structural features of the UEV domain of Tsg101 on which this review will focus. In addition to the vestigial active site region, the N-terminal UEV domain is distinguished from canonical E2 enzymes and from Mms2, another well-studied UEV protein, by an additional N-terminal helix, an extended β-hairpin that links β-strands 1 and 2 and by the absence of the two C-terminal helices normally found in E2 enzymes [17] (Figure 1A, sites 3, 1, 2 and 4, respectively). The hydrophobic cleft exposed by the absence of the C-terminal helices forms the pocket in which P(T/S)AP-containing peptides bind (panel 1B). Mutations in the pocket block recruitment of Tsg101 by HIV-1 Gag, as well as proteins encoded by other viruses containing the motif, and by Hrs, the cellular factor in the ESCRT-0 complex that contains PSAP and two additional motifs, PSGP and PSMP [18,19]. It should be noted that Vps23, the yeast ortholog of the mammalian Tsg101 protein, possesses a PSDP motif that binds elsewhere [20]. It is unclear whether *h*Tsg101 conserves this site; the Ala residue in the motif is a critical determinant of binding for most, if not all, partners. Mutations at the β-hairpin that binds mono- and di-Ub non-covalently (site 2, panels 1C and F) reduce the affinity of Gag-Tsg101 interaction [17] and prevent Gag recruitment of Tsg101 to assembly sites located on the plasma membrane [21]. Co-expression of Gag with Tsg101 bearing a disrupted β-hairpin impairs egress significantly [22]. In addition, small molecules that bind nearby (panel 1D) and disrupt Ub-binding at the β-hairpin interfere with delivery of both Gag and Tsg101 to virus assembly sites on the plasma membrane [23,24]. Interestingly, these small molecules interfere with a broad spectrum of enveloped viruses [24]. Budding of several of these susceptible viruses is Tsg101-independent, indicating that the Ub- and P(T/S)AP- binding functions of Tsg101 are not necessarily linked. An example is the nuclear egress of herpes simplex virus (HSV) types 1 and 2 [25], where the involvement of the protein is not critical [9] (reviewed in [26]). The structurally unique N-terminal helix (site 1) houses critical determinants that recognize tRNA (panel 1E; [15]) and one of the sites occupied by the proximal domain of di-Ub moieties (proximal site-1, Strickland submitted, panel 1G). The distal domain of di-Ub (panel 1F) fits the configuration of the mono-Ub- within the β-hairpin exactly (cf., panel 1C). The proximal domain of di-Ub can alternatively occupy the vestigial active site (site 3; panel 1H, [21].

Interestingly, while mutations at the UEV tRNA-, Ub-, and P(T/S)AP- binding sites all interfere with HIV-1 Gag assembly, of these, only disruption of the P(T/S)AP-binding pocket impairs Hrs-Tsg101 recruitment, indicating that viral- and cellular- mediated protein interactions are subject to different controls. Thus, it appears that HIV-1 and other viruses not only mimic the structural feature of Hrs that permits Tsg101 engagement (i.e., encoding P(T/S)AP-motifs), as demonstrated previously [27] but, in addition, engage the Tsg101 structural features involved in Ub signaling and thereby facilitate the viral assembly process. This review will focus on the recently identified interactions of the UEV domain and their role in virus assembly and budding.

## 2. UEV Structural Features Regulate Tsg101 Recruitment by Its P(T/S)AP-Binding Partners

The N-terminal UEV domain of the Tsg101 protein (aa 1–145) is connected to a central extended coiled-coil (aa~240–311) and a C-terminal helical domain (~330–390) through a Pro-rich linker region (~aa 146–215) (reviewed in [11]). The unique structural features of the UEV domain that houses the pocket where the P(T/S)AP motif is recognized have been described previously [19,28,29]. The domain binds a HIV-1 Gag-p6 peptide with moderate affinity under physiological conditions (Kd = 27 ± 5 µM) [1]. A nonapeptide from a sequence within p6 [PEP_7_(T/S)AP_10_PEE] binds with the same affinity, indicating that the motif and flanking sequences suffice for binding. The core of this nonapeptide (P(T/S)AP, designated as the Late (L) domain in Gag [30] and other viral proteins, is not sufficient for binding, however, these core sequences provide the most important contacts to the pocket [19,28,29]. Direct fusion of Ub to p6 enhances the binding affinity over that of p6 or Ub alone, indicating that the Ub and PTAP binding sites are distinct and, moreover, can be simultaneously occupied. The sites appear to be independent, however as described below and elsewhere, mutations in determinants of Ub binding can weaken, disrupt or, alternatively, enhance the Gag-Tsg101 interaction, even though the P(T/S)AP-binding pocket is intact [15]. CPMG relaxation dispersion NMR, a tool to detect potential conformational exchange and communication between two distal binding sites, suggests that the P(T/S)AP binding pocket and the vestigial active site are coupled dynamically [21].

The UEV domain conserves the general fold of canonical Ub conjugases (E2 enzymes) and of Mms2, another UEV protein whose structure is known [17,28,29,31,32,33]. In both cases, the structural similarity is greatest around the central active site region (aa 53–138 in the Tsg101 UEV) and the regions can be superimposed. As the active site Cys residue in E2 enzymes is replaced in the Tsg101 sequence with a Tyr residue that is highly conserved, it was proposed that Tsg101 might regulate the ubiquitination of short-lived gene products in the manner of a dominant-negative regulator [12,13]. We therefore reasoned that comparison of structures of the Ub-bound UEV with the Ub-bound E2, which were not available at the time the proposal was made, should indicate if the partner Ub moieties are similarly or differently oriented in the complexes and thereby provide clues to support or negate the idea.

**Figure 2:**
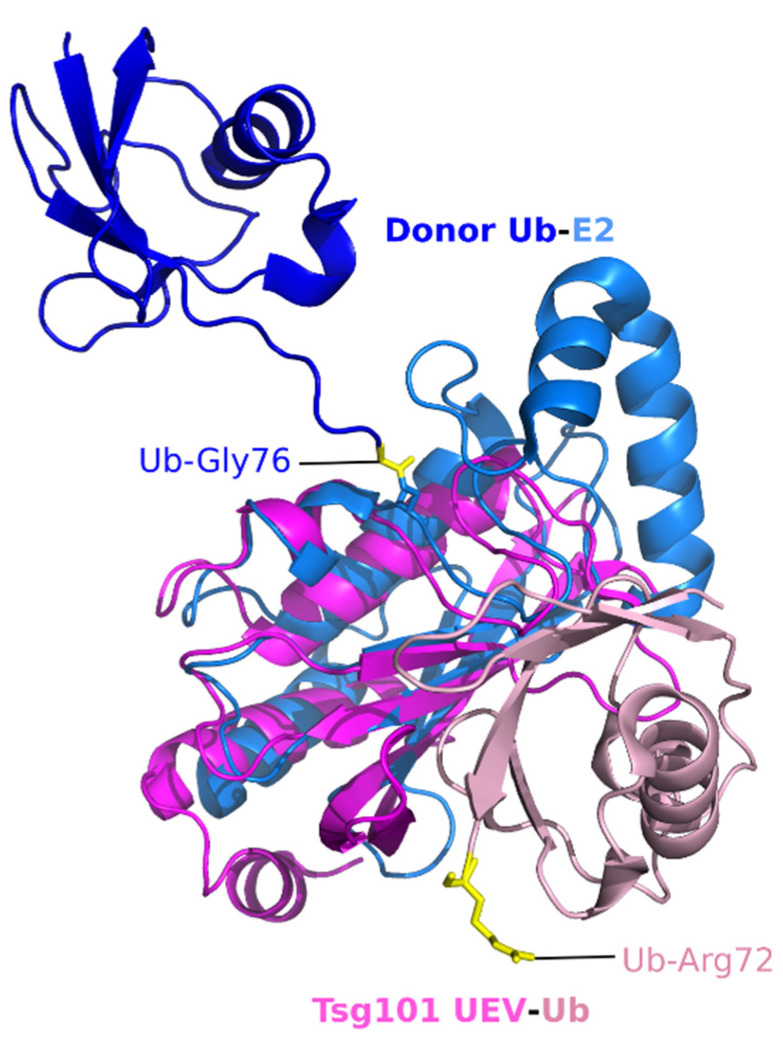
Superimposition of the *h*Tsg101 UEV domain complexed to Ub and canonical E2 (UbcH5) complexed to Ub. Tsg101 is dark pink, the complexed Ub is a light pink; E2 is light blue, the complexed Ub molecule is dark blue. UEV-Ub, PDB ID: 1S1Q [32]; E2-E3-Ub (E3 not shown), PDB ID: 3JVZ [34]. The donor Ub Gly76 residue thioesterified to the E2 is yellow. The C-terminus of the Ub moiety partnered with the UEV is flexible and therefore not apparent in the crystal structure. The last ‘rigid’ residue, R72, is highlighted to give the reader an idea of where the C-terminus lies.

We overlaid the UEV structure onto the crystal structure of the UbcH5 in complex with Ub. As shown in Figure 2, our alignment of the structures (PDB 1S1Q, PDB 3JVZ, [34]) shows that the Ub-binding surface on the Tsg101 UEV is in proximity to the previously characterized Ub binding sites on the E2, although it is distinct. Moreover, the affinities for Ub are in the same order of magnitude (UEV, ~500 μM [17,32], e.g., E2, UbcH5 ~300 μM [35]), thus supporting the possibility that the structural similarity aligns with functional mimicry. However, determining whether a structural basis exists for the hypothesis is challenging. While neither the E2 nor the Ub component undergoes conformational change upon interaction, Ub can adopt many positions relative to the E2, as revealed by comparisons of E2∼Ub structures (reviewed in [36]). It is not clear whether this reflects differences in levels of enzyme activity, crystal-packing effects, or both. Additionally, NMR studies indicate that conjugated Ub is flexible relative to the E2 and that the two proteins behave as two loosely connected entities [37,38]. It also may be significant that in our overlay of the Ub bound to the Tsg101 UEV (UEV: pink, Ub: lighter pink in Figure 2), the most C-terminal proximal residue apparent in the structure, Arg72 (yellow), points away from the UEV. In contrast, in the Ub bound to the overlaid E2, the C-terminal residue, Gly76, is oriented towards the E2 center (Gly76, yellow, shown thioesterified to the E2). Collectively, these considerations suggest that a simple E2-UEV replacement model is unlikely. As discussed below, the fact that small molecules that disrupt the UEV-Ub interaction differentially inhibit constitutive and ligand-induced trafficking of the epidermal growth factor receptor (EGFR) [23] suggests that Ub binding at this site might facilitate interactions pertinent to signaling in distinct transport networks.

**Figure 3:**
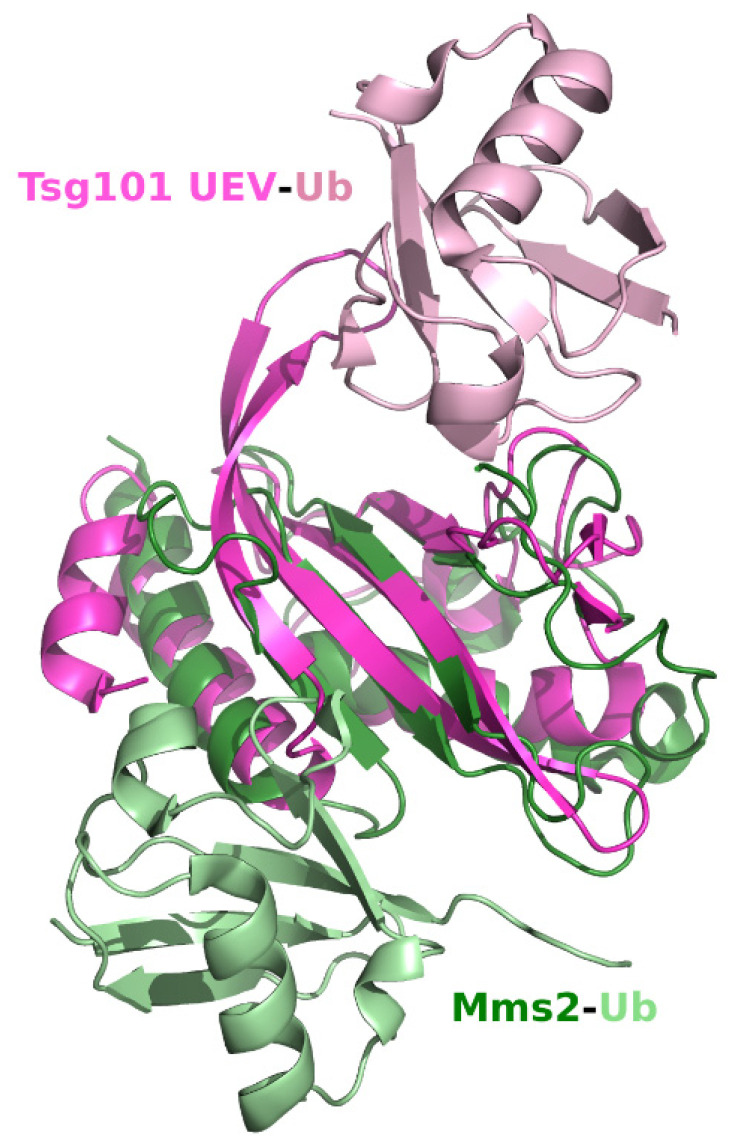
Superimposition of the *h*Tsg101 UEV domain complexed to Ub and *h*Mms2 complexed to Ub. Tsg101 is pink, Mms2 is green and the two Ub molecules in each PDB are lighter versions of the same colors. UEV-Ub, PDB ID: 1S1Q [32]; Mms2-Ub, PDB ID: 1ZGU [46].

The E2 active site Cys residue replaced with Tyr in the Tsg101 sequence is replaced with Asp in the UEV *h*Mms2 [39,40]. Its Ub-binding affinity (100 μM, [41]) is greater than that determined for Tsg101 UEV Ub binding (~500 µM). An overlay of the human Mms2 (*h*Mms2) and Tsg101 UEV (Figure 3) indicates that Ub binds in a very different site on each. The Mms2 site is not equivalent to the previously described mono-Ub binding site nor are either of the regions occupied by the proximal domain of di-Ub (Figure 1G,H and described below). Nevertheless, there is a strong link regarding Lys63 sensing and regulation for the UEV proteins: (*i*), *h*Mms2 forms a complex with the E2 Ub conjugase Ubc13 that functions with the E3 ring-type ligase Rad6 in assembly of Lys63-linked polyUb chains for DNA repair [42,43]; (*ii*), UEV-1, another mammalian homologue of the yeast Mms2 UEV protein, binds Ubc13 forming a heterodimer that also functions in the synthesis of Ub chains linked through Lys63 but not for Ubc13-mediated DNA repair [44,45]; and (*iii*), as noted above, the Tsg101 UEV binds Lys63-linked di-Ub non-covalently but also is not known to function in DNA repair.

Thus, it seems likely that Mms2, UEV-1 and Tsg101 UEV function in analogous manners, i.e., as regulatory subunits of the E2 and E3 enzymes with which they pair in order to increase the functional diversity and Ub conjugation selectivity of the enzyme. Tsg101 associates with both ring-type E3 ligases and with members of the HECT family of Nedd4 E3 Ub ligases (e.g., [47,48,49,50,51]). Nedd4-2 (also designated as Nedd4L), which HIV-1 Gag binds via the adaptor protein AMOT [52] and which was shown to exhibit Tsg101-dependent rescue of mutants lacking the P(T/S)AP motif [53,54], utilizes UBE2D (UbcH5) and UBE2L3 (UbcH7) Ub-conjugating (E2) enzymes to transfer a single tetra-Ub Lys63-linked chain [55]. The Nedd4-2 stimulation of the Gag mutant resulted in ubiquitylation of several ESCRT-I subunits, including Tsg101, suggesting that Nedd4-2 and possibly other Nedd4 isoforms, acting upstream of or together with Tsg101, ubiquitinate and thereby activate ESCRT-I to function in virus budding [53]. Interestingly, however, although Tsg101 was found to be required for the Nedd4-2-mediated release, neither its β-hairpin Ub-binding function nor its PTAP binding function were required. It should be noted that (i), the ESCRT adaptor protein ALIX also strongly stimulates the release of infectious HIV-1 encoding a disrupted PTAP motif [53,56]; (ii), small molecules that bind the UEV domain near the β-hairpin disrupt Ub binding [23] inhibit egress of the ΔPTAP mutant but do not prevent the ALIX-mediated rescue [24]. Thus, both Alix and Nedd4 isoforms can rescue viral budding and Ub-binding at the UEV β-hairpin is not required in either case.

We suspect that Tsg101 recruits de-ubiquitinating (DUb) enzymes that contribute to its regulation of the E2/E3 enzyme: Impairing or abrogating Tsg101 interaction by mutating the P(T/S)AP motif in HIV-1 Gag results in polyubiquitination of Gag [57,58]. Tsg101 Ub-binding at the β-hairpin could thus function in regulation of DUb rather than ligase activity, at least in some cases. However, it should be noted that, in the context of a minimal Gag protein, the presence of the PTAP motif enabling Tsg101 binding increased Gag ubiquitination [59], consistent with Tsg101 regulation of an associated ligase. Ubiquitination of Gag has also been found to be highly dependent on membrane association [60]. Thus, a combination of factors, opposing and synergistic, might contribute to the manner in which Tsg101 functions with associated Dubs, ligases and their substrates.

## 3. Small Molecule Inhibition Implicates Non-Covalent Ub-Binding at the Tsg101 β-Hairpin in Trafficking of HIV-1, EBV, and HSV-1/2 Capsid Proteins to the Cell Periphery

Tsg101 provides unique opportunities for novel drug development. Targeting of a required host factor rather than a viral-encoded protein is anticipated to minimize the emergence of resistant viruses. It also provides an opportunity to obtain broad-spectrum inhibitors since, as noted above, many human pathogens require Tsg101. Another unique advantage, at least in the case of viruses like HIV that bud from the plasma membrane, is that blocking its action results in the accumulation of particles on the cell surface, thereby improving antigenic recognition of infected cells. This might have an added supportive effect of helping the immune system to identify and remove infected cells and, in this way, complement natural innate and adaptive immunity mechanisms more readily. The UEV domain is the only region of the protein for which structural information is available at the resolution required for drug design. Most efforts targeting Tsg101 have focused primarily on the PTAP site in that domain and have led to the identification of both cyclic [61,62,63] and multidentate [64,65,66,67] peptide and small molecule inhibitors [24,25,68]. Interestingly, while peptide inhibitors targeting PTAP-dependent Gag budding had no effect on release of a PTAP mutant [61], the small molecule inhibitors targeting UEV Ub-binding inhibit release irrespective of PTAP engagement [23]. This raises the possibility that combinatorial therapies might be feasible.

As a critical component of the ESCRT pathway involved in the recognition of ubiquitinated cargo for delivery to MVBs for endolysosomal degradation [69], Tsg101 has been used to assess the role of ESCRT machinery in down-regulation of several plasma membrane-associated proteins by diverse cellular Ub ligases, including MARCH8 [52,70,71]. Under conditions of MARCH8 over-expression, the transmembrane protein CD98 was Ub-modified and diverted from trafficking to the plasma membrane to late endosomes, where it was ultimately degraded [51]. Tsg101 depletion prevented the MARCH8 intervention. The ubiquitylation of CD98 by MARCH8 was reversed by Ub-specific protease 6 (USP6) and dependent on its deubiquitylating activity [68]. The DUb was also capable of counteracting the effect of MARCH ligases on the recycling of CD44, CD147 and, to a lesser degree, MHC-I [51,72]. Collectively, these findings support the possibility, raised above, that Tsg101 participates with E3 ligases and DUbs in regulating ubiquitylation and deubiquitylation events necessary for trafficking and sorting of cellular cargo.

**Figure 4:**
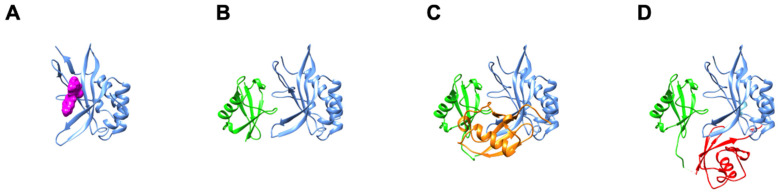
UEV binding modes of tenatoprazole compared to mono- and di-Ub. Panel (**A**), Tsg101 UEV, blue; tenatoprazole, magenta; PDB ID: 5VKG [23]. Panel (**B**), Tsg101 UEV, blue; mono-Ub bound at the β-hairpin, green; PDB ID: 1S1Q [32]. Panel (**C**), Tsg101 UEV, blue; di-Ub distal domain, green, bound at the mono-Ub binding site, di-Ub proximal domain-1, orange, bound at the N-terminal α-helix-1 site; PDB ID: 6UD0 (Strickland et al., submitted). Panel (**D**), Tsg101 UEV, blue; di-Ub distal domain, green, bound at the mono-Ub binding site, di-Ub proximal domain-2, red, bound at the vestigial active site; PDB ID: 6UD0.

The identification of tenatoprazole and an NMR structure of the complex [23] provided important “proof-of-concept” that regions in the UEV domain outside of the PTAP-binding pocket could be exploited for discovery of anti-viral agents. Prazoles are small molecules that bind the UEV domain near the β-hairpin and disrupt Ub binding there ([23]; Figure 4). As noted above, they interfere with the ability of HIV-1 Gag to recruit Tsg101 to the plasma membrane assembly site and with the ability of EBV and HSV to mobilize the factors required for immature capsid formation and egress from the nuclear membrane assembly site [25,70]. Through siRNA-mediated depletion/replacement experiments, residue Cys73 in Tsg101 was demonstrated to be the prazole target in the case of HIV-1 and EBV. In the latter case, the amino-terminal domain of the large tegument protein VP1/2 functions as a ubiquitin specific protease (DUb) that is highly conserved throughout the herpesvirus group [71] (BPFL1, in the case of EBV) and whose activity is critical for recruitment of the Tsg101 protein to the nuclear rim [73]. A PSAP motif in the domain contributes to the recruitment. Thus, the similarities in requirements for recruitment of Tsg101 to spatially distinct membranes within the cell exhibited by these unrelated viruses implicate the UEV Ub-binding function of the Tsg101 protein in cargo recognition and sorting for endocytic trafficking independent of its role as conduit to the downstream ESCRT-II and -III complexes.

As noted above, under conditions where prazoles inhibit viral particle production, they also inhibit the constitutive recycling of EGFR to the plasma membrane [23]. Interestingly, several cellular proteins whose trafficking to the cell periphery is controlled by Tsg101, the MARCH8 Ub ligase and the DUb USP6, e.g., CD44, CD98 and CD147 are all packaged into HIV-1 virions [74]. These findings suggest that HIV-1 Gag exploits a cellular trafficking pathway for delivery to the cell periphery that is controlled by Tsg101, Ub ligases and DUbs. We speculate that herpesviruses employ a similar strategy, directed through their VP1/2 structural equivalents. In all cases, encapsidation of the co-routed cellular proteins could be adventitious or vital to viral infectivity. As already noted, under conditions where prazoles inhibit Gag, Tsg101, and EGFR delivery to the periphery, no inhibition of ligand-induced EGFR down-regulation was detected [23]. The differential inhibition observed here and described above suggests that prazoles distinguish the signals that tag molecules for down-regulation in degradative compartments versus transport to the plasma membrane versus delivery to the nuclear membrane and that UEV non-covalent Ub-binding is a component of such signaling. The Ub-UEV poses captured in structural analyses might reflect the range of possible interactions of the putative UEV regulatory subunit with E2, E3 and DUb enzyme partners.

## 4. The Vestigial Active Site Region Participates in Lys63-Linked Di-Ub Binding

**Figure 5:**
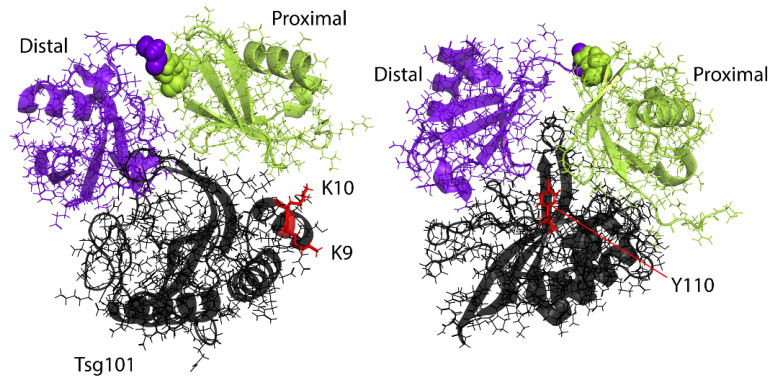
Binding modes of Lys63-linked di-Ub on the Tsg101 UEV domain. (**Left**), Di-Ub complex with proximal domain in contact with N-terminal region; (**Right**), Di-Ub complex with proximal domain in contact with vestigial active site. Key determinants of recognition of the two binding modes (proximal-1, K9, K10; proximal-2, Y110, respectively) are highlighted in red sticks. PDB ID: 6UD0.

Supporting the possibility that differential prazole susceptibility reflects Ub signaling features, we tested for and found that, in addition to binding mono-Ub, the Tsg101 UEV domain recognizes several forms of diubiquitins (di-Ub), with a preference for Lys63-linked di-Ub (Figure 5, [21]. The previously identified mono-Ub binding site accommodates the distal domain of di-Ub, while the proximal domain alternatively binds two different sites, the vestigial active site that defines the UEV and the Tsg101 N-terminal helix. There is rapid exchange between the two sites. The substitution of Ala for residues at each site had opposite impact on the Gag-Tsg101 interaction: Mutations in the vestigial active site prevented Tsg101-interaction with HIV-1 Gag and, thereby, the recruitment of Tsg101 to viral assembly sites at the cell periphery. This outcome was unexpected as the spatially distinct P(T/S)AP-binding pocket in the Tsg101 protein (cf., Figure 1, compare panels B and H) was intact. However, as noted above, we have shown that these sites appear to be dynamically linked. In contrast to the effect of the vestigial active site mutations, the substitution of Ala for the Ub- and RNA- binding determinants on the N-terminal helix (cf., Figure 1, panels E and G) did not impair binding to Gag [15]. Indeed, the interaction was increased, Tsg101 recognition of Gag-Ub was enhanced, and Gag-Tsg101 complexes accumulated in the cell interior rather than at the plasma membrane. This outcome, also unexpected, revealed that Tsg101 possesses determinants that control the translocation destination. Interestingly, the ability of Hrs to recruit Tsg101 to early endosomes was unaffected by mutations at either Tsg101 UEV location (Strickland submitted). Thus, Hrs-PSAP, HIV-1 Gag-P(T/S)AP, HSV VP1/2-PSAP and EBV BPFL1-X(T/S)XP are most likely recognized differently and influenced by additional determinants in each protein. In the case of Hrs, the PSAP motif was reported to be sufficient for Tsg101 recruitment [18], however, several additional determinants of the interaction have been identified [19]. This most likely explains why UEV mutants impaired in PTAP motif binding were unable to rescue HIV-1 budding but did rescue downregulation of endogenous EGF receptor [75]. Based on biochemical/genetic studies [19] and prazole susceptibility ([23], UEV Ub-binding is not important for Hrs-Tsg101 interaction. Intriguingly, however, based on prazole sensitivity, it appears to be a key factor for productive recruitment by the viral proteins.

**Figure 6:**
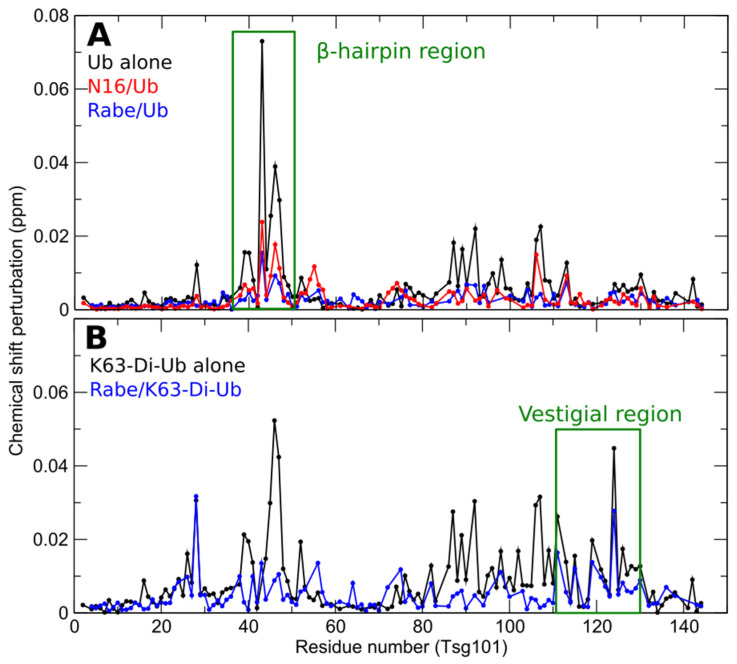
Prazoles block UEV binding of mono- but not di- Ub. Panel (**A**) Chemical shift perturbations of Tsg101 upon addition of ubiquitin, either alone (1:1 ratio–black), or when covalently precomplexed with tenatoprazole (N16, red) [23] or rabeprazole (blue). Panel (**B**) Chemical shift perturbations of Tsg101 upon addition of Lys63-linked di-Ub alone (black) or after first complexing with rabeprazole (blue).

The fact that the distal domain of di-Ub exactly fits the space occupied by mono-Ub in the X-ray structure of the Tsg101 UEV-Ub complex suggests that mono- and di- Ub share the sites rather than occupy overlapping sites. To address this question experimentally, we determined the effect of prazole-mediated disruption at the β-hairpin. If independent, prazole binding should not preclude di-Ub binding. In contrast, if the sites are shared, prazoles should prevent both mono- and di-Ub binding. As shown in Figure 6, the UEV residues perturbed by binding of mono-Ub (panel A, black line) and Lys63-linked di-Ub (panel B, black line) overlap considerably in the region of the β-hairpin. However, di-Ub perturbs residues elsewhere in addition. Mono-Ub binding was effectively eliminated by tenatoprazole (N16) and rabeprazole (Rabe) [23,24] (panel 6A, red line and blue line, respectively). Rabeprazole, was slightly more effective than tenatoprazole, but nevertheless failed to dampen binding of di-Ub in the region of the vestigial active site and elsewhere (panel 6B, blue line). EGFR down-regulation which, as noted above, we found to be prazole-resistant, has been reported to require Lys63-linked di-Ub modification [76]. Finding that prazoles differentially inhibit binding of mono- and di-Ub to the UEV domain supports the conclusion that these small molecules target Tsg101-related functions very specifically and will be useful probes to distinguish functions of the Tsg101 protein that are controlled by mono- vs. di-Ub binding.

## 5. Prazole Resemblance to Nucleic Acid Bases Suggest the UEV Recognizes RNA

The structural similarity of prazoles to nucleotides prompted us to consider the possibility that the UEV domain of Tsg101 might recognize nucleic acid. Indeed, we and others have observed that Tsg101 is pulled-down with the HIV-1 nucleocapsid protein (NC) from cell lysates and in vitro in the presence of RNA [3,77]. Moreover, factors in ESCRT-II, ESCRT-III, and the ESCRT adaptor Alix have also been reported to interact with nucleic acids [78,79,80,81,82]. Collectively, these observations encouraged the notion that Tsg101 might bind RNA directly. As interactions with tRNA constitute the most frequent binding event between cytosolic Gag and RNA [83], we tested a commercial mixture of yeast tRNA and identified UEV residues undergoing chemical shift perturbances on the face opposite to that containing the mono-Ub-, P(T/S)AP- and prazole-binding regions as well as on the same face (cf., Figure 1E, [15]). Some determinants of tRNA recognition were also critical for recognition of di-Ub proximal domain-1 (e.g., Lys9, Lys10) or di-Ub proximal domain-2 (e.g., Tyr110), making it unlikely that RNA and di-Ub might bind the same Tsg101 molecules. In any event, the RNA binding affinity is expected to be significantly higher. The substitution of Ala for any of these residues altered the Tsg101 interaction with Gag supporting the conclusion that both RNA recognition and di-Ub binding at both sites are important. In contrast, Tsg101- interaction with Hrs was not affected by mutation of any of the sites. Attempts using RNA Bind-n-Seq (RBNS) [84] to determine if UEV recognition was based on a specific subset of RNA and to obtain a quantitative assessment of the binding provided no evidence for sequence-specific binding to a complete library of RNAs 40 nucleotide in length [15]. Interestingly however, we observed that mutation of a residue specific to UEV recognition of tRNA, His115, was deleterious to Tsg101 interaction with Gag but not p6.

This finding suggests that UEV-RNA interaction is important for Tsg101 recognition by a region in Gag outside of the p6 domain and is reminiscent of the facilitating role in budding of the NC domain in Gag in the absence [85] or presence [15,86] of the intact p6 domain. Indeed, deletion of the NC zinc fingers (ZnF) decreases the amount of Gag-Tsg101 interacting complexes in cells and we and others have demonstrated that budding of Gag mutants lacking these elements in NC was rescued by providing Tsg101 at the Gag assembly site (i.e., as Gag-ΔZnF-Tsg101 [15,77]). These findings are supported by NMR data showing chemical shift perturbations in the NC domain in and outside of the zinc finger elements upon Tsg101 binding [87]. The structural elements in NC that Tsg101 can functionally replace [15] are required for RNA binding [88]. Thus, although the identity of the RNA that is recognized by Tsg101 in the cellular setting is not known at this time, it is likely that binding to this RNA species permits the NC domains to cooperate with p6 in recruitment of the Tsg101 protein.

In a broader context, it is known that several P(S/T)AP-containing proteins that bind to Tsg101 function in RNA processing, silencing, vesicular trafficking from the endoplasmic reticulum, and transcriptional regulation [89]. Additionally, dozens of E3 ligases have RNA binding functions [90]. As suggested for Tsg101, this partnering links some RNA-dependent process with protein ubiquitylation. Tsg101 is secondarily linked to the E3 enzyme, Mid1, through the abscission process in cytokinesis where both participate [91]. Moreover, Rsp5, the *Saccharomyces cerevisiae* homolog of the mammalian E3 ligase Nedd4, can regulate RNA-dependent processes despite not binding directly to nucleic acids. Perhaps Tsg101, which binds Nedd4 [49], provides this function if Nedd4 can mediate those events.

## 6. Summary and Future Perspectives

Recent studies have revealed that the N-terminal UEV domain of the Tsg101 protein recognizes tRNA and at least three different di-Ub molecules (Lys48-linked di-Ub, Lys63-linked di-Ub and N-linked di-Ub), with a preference for the Lys63-linked form, in addition to the mono-Ub and peptides bearing P(T/S)AP motifs discovered decades ago. The precise role in viral egress of these non-covalent binding functions has not yet been established and the relationship between them is not yet clear. However, we have learned that the UEV domain is recognized by small molecules whose binding interferes with the mono-Ub binding function and, consequently, viral egress. Released viral particles are not infectious, indicating that the imposed defect impacts a post-maturation event important for the next round of replication. Several questions arise: Why does mutation of the Tyr residue in the UEV vestigial active site that is conserved in Tsg101 orthologues and substitutes for the active site Cys residues in E2 enzymes site impair Gag-Tsg101 interaction, especially since the P(T/S)AP-binding pocket is intact under these conditions [3]? How does the N-term α-helix in the Tsg101 protein, which can recognize RNA and di-Ub, influence recognition of Gag when Gag is modified by covalent addition of Ub? Is the recognition of Ub-modified Gag determined by residues in the N-terminal α-helix linked to the observed change in subcellular localization of Gag-Tsg101 and the apparently enhanced Gag-Tsg101 binding when the residues are mutated? What influences delivery of the Tsg101-viral protein partner complexes to plasma membrane versus nuclear membrane locations? In the case of the herpesviruses, the presumptive partner is a virally encoded DUb whose catalytic activity is critical [73]. To date, no DUb counterpart has been identified for HIV-1 Gag but DUb participation seems likely [92,93].

These questions are as yet unanswered, but our studies indicate that the structural features that distinguish the Tsg101 UEV from canonical E2 enzymes and Mms2 provide unique Ub- and RNA-binding surfaces that underlie the protein’s ability to facilitate virus production. Interestingly, like the Tsg101 UEV domain, the non-canonical E2 enzyme Ube2c (UbcH10) possesses a unique N-terminal extension that is thought to provide this E2 enzyme with specificity towards substrates. Similarly, N-terminal extensions of members of the Ube2e family (UbcH6, Ube2e2 and UbcM2) are implicated in the enzymes’ substrate interactions [94] although functional consequences have not yet been resolved. We have shown that the unique N-terminal extension on the Tsg101 UEV contains determinants that recognize novel RNA and di-Ub binding partners that influence both HIV-1 Gag localization and Ub modification state recognition [15]. Possibly, just as recognition of Tsg101-P(T/S)AP binding by viral and cellular proteins added to our understanding of the mechanistic underpinning of the critical ESCRT cellular machinery, similarly, understanding the roles of the other unique structural features of the Tsg101 protein may provide new insight into functions important for both cellular operations and viral replication. Discovery of the di-Ub binding and RNA interactions followed identification of the Tsg101 UEV as a target for covalent attack of sulfonamides, active forms of prazole prodrugs used widely for treatment acid of reflux disease [95]. These compounds exhibit promise as inhibitors of the replication of a broad spectrum of unrelated enveloped viruses [24], making them potential candidates for alternative indication development as antiviral agents as well as basic research tools for investigation of still-to-be elucidated Tsg101 functions in viral replication.

## Data Availability

Not applicable.
